# Weekly practice frequency of individual yoga components and subjective wellbeing in Hungarian yoga practitioners: a nationwide cross-sectional online survey

**DOI:** 10.3389/fpsyg.2026.1941678

**Published:** 2026-10-28

**Authors:** Gusztáv József Tornóczky, Sunil Singh Yadav, Henriett Nagy, İsmail İlbak, Vera Klier, Dorottya Bosnyák, Attila Szabo, Réka Erika Kovács

**Affiliations:** 1 Institute of Health Promotion and Sport Sciences, Faculty of Education and Psychology, ELTE Eötvös Loránd University, Budapest, Hungary; 2 Physical Education and Exercise Centre, Medical School, University of Pécs, Pécs, Hungary; 3 School of Naturopathy and Yogic Sciences, SGT University, Gurugram, India; 4 Department of Personality and Health Psychology, Institute of Psychology, ELTE Eötvös Loránd University, Tartu, Estonia; 5 Faculty of Sport Sciences, İnönü University, Malatya, Türkiye; 6 Doctoral School of Education, Faculty of Education and Psychology, ELTE Eötvös Loránd University, Budapest, Hungary; 7 Department of Psychology and Health Management, Faculty of Health and Sport Sciences, Széchenyi István University, Győr, Hungary

**Keywords:** mental health, subjective wellbeing, yoga, yoga components, yoga practice

## Abstract

**Background:**

Yoga is a multidimensional mind–body practice, yet little is known about whether the weekly practice frequency of its individual components is differentially associated with subjective wellbeing (SWB). This study examined the associations between the weekly practice frequency of asana, pranayama, relaxation, and meditation and SWB among Hungarian yoga practitioners.

**Methods:**

A cross-sectional online survey based on convenience snowball sampling was conducted in April–May 2021, during the COVID-19 pandemic, among 379 adult yoga practitioners (44.31 ± 10.12 years). SWB was assessed using the World Health Organization-Five Wellbeing Index (WHO-5). Associations between participant characteristics and SWB were examined using one-way ANOVA and Pearson’s correlations. Separate ANCOVA models examined associations between the weekly practice frequency of each yoga component and SWB after adjustment for place of residence, other physical exercise, yoga practice duration, and yoga expertise (Holm–Bonferroni corrected). Independent associations were examined using multiple linear regression, with a dummy-coded sensitivity analysis.

**Results:**

Place of residence, yoga expertise, other physical exercise, and, weakly, yoga practice duration were associated with SWB. After adjustment, all four yoga components remained significantly associated with higher SWB (corrected *p* ≤ 0.029), with medium effect sizes for asana (ηp^2^ = 0.070) and meditation (ηp^2^ = 0.061) and small effect sizes for pranayama (ηp^2^ = 0.050) and relaxation (ηp^2^ = 0.024). Daily practice was consistently associated with the highest SWB. The regression model explained only 12.9% of the variance in SWB (Cohen’s *f*^2^ = 0.15), and only asana (*B* = 0.69, 95% CI [0.22, 1.17]) and meditation frequency (*B* = 0.61, 95% CI [0.21, 1.00]) remained independently associated with SWB, as supported by the sensitivity analysis.

**Conclusion:**

In this predominantly female, highly educated convenience sample, more frequent weekly practice of all four yoga components was associated with higher SWB. However, after accounting for participant characteristics, yoga experience, and the overlap among components, only asana and meditation frequency remained independently associated with SWB. These associations were small, and most of the variance in SWB remained unexplained. Given the cross-sectional design, causal conclusions cannot be drawn and generalization should be cautious; longitudinal and intervention studies of individual yoga components are needed.

## 1 Introduction

### 1.1 Subjective wellbeing and mental health

Subjective wellbeing (SWB), encompassing individuals’ cognitive evaluations of their lives together with their positive and negative affect, is a central construct in positive mental health research and can be reliably assessed using well-validated instruments such as the World Health Organization Five Wellbeing Index (WHO-5) (Topp et al., 2015). A growing body of evidence indicates that higher levels of SWB are consistently associated with better physical and mental health, healthier lifestyles, and greater longevity, highlighting its importance as both a psychological resource and an important indicator of public health (Diener et al., 2017).

Furthermore, higher levels of SWB have consistently been associated with lower levels of psychological distress, including depression and anxiety symptoms, perceived stress, loneliness, insomnia, and suicidal ideation, as well as with greater life satisfaction, resilience, meaning in life, health-related quality of life, and perceived mental health across diverse populations and cultural contexts (Domenech et al., 2025; Iversen et al., 2026; Sischka et al., 2020, 2026; Topp et al., 2015).

Beyond its established importance as an indicator of positive mental health, higher SWB has been linked with greater psychological resilience across diverse populations, suggesting that individuals with higher wellbeing may be better equipped to adapt and to cope with everyday challenges and adversity (Aliyev et al., 2024; Chow et al., 2018; Rayani et al., 2024).

Given the central role of SWB in positive mental health and its close associations with multiple indicators of psychological functioning, identifying modifiable lifestyle factors that may enhance wellbeing has become an important focus of contemporary health and behavioral research. Among these, yoga has emerged as one of the most extensively studied mind–body practices for promoting mental health and psychological wellbeing, supported by a substantial and continuously expanding body of scientific evidence (Park and Slattery, 2021). Recent reviews have identified more than 120 yoga interventions targeting mental health and wellbeing across 24 countries, highlighting both the global research interest and the broad applicability of yoga in diverse populations (Frazier et al., 2026). Consistent with this growing evidence base, multiple systematic reviews have reported beneficial associations of yoga with perceived stress, anxiety, depression, and psychological wellbeing, further supporting its role as a promising lifestyle approach for mental health promotion (Frazier et al., 2026; Malipeddi et al., 2024).

### 1.2 Yoga, mental health and subjective wellbeing

The available evidence suggests a positive association between yoga practice and SWB, although the strength of this association appears to vary according to the populations studied, intervention characteristics, comparison groups, and wellbeing measures applied (Hendriks et al., 2017; Takemura et al., 2025; Wadhen et al., 2025). Nevertheless, previous yoga reviews have primarily synthesized broader wellbeing constructs (Hendriks et al., 2017), whereas SWB assessed with the WHO-5 has been investigated less frequently.

Meta-analytic evidence indicates that the strongest psychological benefits of yoga have been observed among older adults. Compared with inactive controls, yoga has been associated with improvements in mental wellbeing, health-related quality of life, vitality, sleep quality, and depressive symptoms (Sivaramakrishnan et al., 2019; Tulloch et al., 2018). In contrast, evidence from healthy adult populations suggests more modest associations, with psychological wellbeing improving primarily relative to inactive controls, whereas differences are less consistent when yoga is compared with other forms of physical activity (Hendriks et al., 2017).

Several complementary psychological and physiological mechanisms have been proposed to explain the positive association between yoga practice and SWB. Current evidence suggests that yoga may promote wellbeing by supporting stress regulation, enhancing mindfulness (Yang et al., 2023) and interoceptive awareness, and strengthening emotion regulation and self-compassion (Garnsey et al., 2026; Park et al., 2020; Pascoe et al., 2021). Additional pathways, including breath-mediated autonomic regulation, spirituality and meaning in life, as well as social connectedness, have also been proposed, although their specific contributions to SWB remain less well understood (Park et al., 2021; Parkinson and Smith, 2023; Tellhed et al., 2019). Taken together, these findings provide a plausible theoretical framework linking yoga practice with higher SWB while also suggesting that different components of yoga may contribute through partially distinct psychological and physiological mechanisms.

### 1.3 Yoga as a multidimensional mind–body practice: components, practice duration, and frequency

Originating from the classical eight-limbed tradition of yoga, contemporary yoga has evolved into a multidimensional mind–body practice that integrates several interconnected yet distinct components (Gupta et al., 2024; Piran and Neumark-Sztainer, 2020). Although traditional yoga encompasses ethical, philosophical, and spiritual dimensions, contemporary yoga research and practice primarily focuses on four core components: physical postures (asana), breathing techniques (pranayama), relaxation, and meditation (Frazier et al., 2026; Mandlik et al., 2023). These components are typically practiced in combination within a single yoga session, yet they represent distinct aspects of yoga practice.

Emerging evidence suggests that the individual components of yoga may contribute differently to psychological functioning and wellbeing, although the available evidence remains heterogeneous and does not support the superiority of any single component (Matko et al., 2021, 2022; Mu et al., 2026). Experimental and mechanistic studies indicate that postures, breathing exercises, relaxation, and meditation may influence partly distinct psychological and physiological processes, including emotion regulation, mindfulness, interoceptive awareness, and autonomic regulation, thereby contributing to different aspects of mental health and wellbeing (Gust and Bryan, 2024; Park et al., 2020; Tellhed et al., 2019; Yadav et al., 2024). Nevertheless, the overall pattern of evidence suggests that these components are complementary rather than competing. Systematic reviews have consistently reported that multicomponent yoga interventions integrating postures, pranayama, relaxation, and meditation generally produce more favorable outcomes for depression, anxiety, stress, mood, and psychological wellbeing than simpler or single-component approaches (Matko et al., 2021, 2022; Saroha et al., 2025). Furthermore, emerging evidence indicates that regular and long-term engagement in individual yoga components is associated with higher levels of wellbeing and related psychological resources (Cseh et al., 2026; Parkinson and Smith, 2023).

Beyond the specific components of yoga practice, both practice duration and practice frequency have been suggested as important determinants of its psychological benefits. However, findings regarding yoga practice duration remain inconsistent. While longer-term practitioners generally perceive yoga as beneficial for their health (Ross et al., 2013), national surveys from the United Kingdom and Germany found that weekly practice frequency, rather than the number of years of practice, was more consistently associated with psychological wellbeing and health-related outcomes (Cartwright et al., 2020; Cramer et al., 2019). It therefore remains unclear whether accumulated years of yoga experience are associated with SWB independently of current practice habits.

In contrast, evidence regarding practice frequency is more consistent. Systematic reviews indicate that more frequent yoga practice is generally associated with better mental health outcomes, with the greatest benefits typically observed among individuals practicing at least three times per week or daily (Brinsley et al., 2021; Cheng et al., 2025; Malipeddi et al., 2024; Upadhyay et al., 2022). A similar pattern has been reported for SWB, with higher weekly yoga practice frequency associated with more favorable SWB outcomes (Tornóczky et al., 2026). Emerging evidence further suggests that more frequent practice of the individual yoga components is associated with higher SWB (Cseh et al., 2026), while regular engagement in the core components of yoga has also been linked to more favorable health outcomes (Cramer et al., 2019). Nevertheless, it remains unclear whether the weekly practice frequency of the individual yoga components is equally associated with SWB, highlighting the need to examine the independent association of each component.

### 1.4 Aims and hypotheses

Against this background, the present study among Hungarian yoga practitioners had three aims: (1) to examine the associations between sociodemographic and yoga-related characteristics and SWB; (2) to examine the associations between the weekly practice frequency of the individual yoga components (asana, pranayama, relaxation, and meditation) and SWB after adjustment for relevant participant and yoga practice characteristics; and (3) to determine whether the weekly practice frequency of the individual yoga components remains independently associated with SWB when all four components are considered simultaneously together with relevant participant and yoga practice characteristics. Based on the available literature, the following hypotheses were formulated:

*H1*: Selected sociodemographic and yoga-related characteristics are significantly associated with SWB among Hungarian yoga practitioners.

*H2*: Higher weekly practice frequency of each individual yoga component (asana, pranayama, relaxation, and meditation) is associated with higher SWB after adjustment for relevant participant and yoga practice characteristics.

*H3*: The weekly practice frequencies of the individual yoga components are independently associated with SWB when all four components are considered simultaneously together with relevant participant and yoga practice characteristics.

## 2 Methods

### 2.1 Participants

An *a priori* sample size calculation was performed using G*Power 3.1.9.7 to determine the minimum sample size requirement for the study’s primary multivariable analysis, namely the multiple linear regression model examining the independent associations of weekly practice frequency of the individual yoga components with SWB. The analysis was conducted using the *F* tests family (Linear multiple regression: Fixed model, *R*^2^ deviation from zero), with eight predictors, an alpha level of 0.05, a desired statistical power of 0.95, and an anticipated small-to-moderate effect size (*f*^2^ = 0.07). Based on these parameters, the minimum required sample size was 333 participants.

The final analytic sample consisted of 379 adult Hungarian yoga practitioners, thus exceeding the required minimum sample size and indicating that the study was adequately powered for the planned primary multivariable analysis. Eligibility criteria were defined *a priori* and required participants to (a) be aged 18 years or older, (b) currently practice yoga at least once per week at the time of the survey, and (c) provide electronic informed consent. Individuals who did not meet these eligibility criteria were not eligible to participate in the study. Participation was entirely voluntary, and only respondents who fulfilled all eligibility criteria and provided informed consent were included in the final sample.

### 2.2 Procedure

The study was conducted as a cross-sectional online survey among Hungarian yoga practitioners between April 17 and May 17, 2021, during the COVID-19 pandemic, at a time when Hungary was emerging from a severe third pandemic wave and restrictions on leisure and sports facilities were only gradually being lifted. The survey had the official support of the Hungarian Yoga Teachers’ Association (HYTA). The study protocol was approved by the Research Ethics Committee of the Faculty of Education and Psychology, Eötvös Loránd University (Approval No. 2021/240), and all procedures were carried out in accordance with the ethical principles of the Declaration of Helsinki.

Participants were recruited nationwide using a convenience snowball sampling strategy. An invitation containing information about the study and a link to the online questionnaire was distributed by e-mail to approximately 280 registered yoga instructors affiliated with HYTA. These instructors were asked to forward the invitation to their current students and to other eligible yoga practitioners within their professional networks, thereby facilitating broader dissemination of the survey across Hungary. Because the number of practitioners who ultimately received the invitation is unknown, a response rate could not be calculated.

Eligibility was assessed at the beginning of the questionnaire, and only respondents who met all inclusion criteria were allowed to proceed to the survey. Data were collected anonymously using a structured online questionnaire administered via Google Forms. No directly identifying personal data were collected. To ensure complete data collection, the survey was configured so that respondents were required to answer all study items before submission; consequently, no missing data were observed in the final dataset.

### 2.3 Assessments

The questionnaire assessed participants’ sociodemographic characteristics, yoga practice characteristics, and SWB, which served as the primary outcome of the study. Specifically, the questionnaire included items on age, sex, educational attainment, occupational status, place of residence, yoga practice duration, yoga expertise (yoga practitioner vs. yoga instructor), participation in other forms of physical exercise, and the weekly practice frequency of the four yoga components examined in this study (asana, pranayama, relaxation, and meditation). Participants were asked to report, separately for each component, how frequently they practiced it during their yoga sessions in a typical week, using four response categories: once a week, 2–3 times a week, 4–5 times a week, and daily. Because these components may be combined within the same yoga session, the four frequency measures were assessed independently and were not mutually exclusive. Each frequency was assessed with a single self-report item, and the classification of practices into the four components relied on participants’ own judgement. No reliability or validity data are available for these single items; the implications of this are considered in the Limitations section. SWB was assessed using the Hungarian version of the World Health Organization-Five Wellbeing Index (WHO-5).

#### 2.3.1 World Health Organization-five wellbeing index (WHO-5)

Subjective wellbeing was assessed using the Hungarian version of the WHO-5 (Bech et al., 1996). The WHO-5 is a five-item self-report questionnaire designed to assess positive mental wellbeing over the preceding 2 weeks. Previous studies have demonstrated its good construct validity as a unidimensional measure of wellbeing and its usefulness for screening depression and evaluating treatment outcomes (Bech et al., 1996; Heun et al., 1999).

The Hungarian version of the WHO-5 was adapted and validated using data from the Hungarostudy 2002 national representative health survey, which included 12,668 adults and was representative of the adult Hungarian population with respect to age, sex, and place of residence (Rózsa et al., 2003; Susánszky et al., 2006). In the Hungarian adaptation, the original six-point response scale was replaced by a simplified four-point Likert scale, with each of the five items scored from 0 to 3 (0 = not at all characteristic, 1 = barely characteristic, 2 = characteristic, 3 = completely characteristic). The five item scores were summed to yield a total score ranging from 0 to 15, with higher scores indicating higher levels of SWB. Because of this modified response format, total scores are not directly comparable with the 0–25 raw score (or the 0–100 percentage score) of the original WHO-5. The Hungarian version has demonstrated good internal consistency reliability (Cronbach’s α = 0.85), and reliability was similarly high in the present sample (Cronbach’s α = 0.87).

### 2.4 Statistical analyses

Descriptive statistics (means, standard deviations, frequencies, and percentages) were calculated to summarize participant characteristics and the weekly practice frequency of the individual yoga components. Prior to the main analyses, the assumptions of parametric statistical procedures were examined. Normality was evaluated using standardized skewness and kurtosis *z*-values according to the recommendations of Kim (2013). Homogeneity of variances was assessed using Levene’s test. For the ANCOVA models, the assumptions of linearity and homogeneity of regression slopes were additionally examined before the analyses. Assumptions of the multiple linear regression model were evaluated by inspecting standardized residuals, the Durbin–Watson statistic, and multicollinearity diagnostics (tolerance, variance inflation factor [VIF], and condition indices).

Associations between categorical participant characteristics (sociodemographic and yoga-related variables) and SWB were examined using one-way analyses of variance (ANOVA), whereas associations between continuous variables (age and yoga practice duration) and SWB were examined using Pearson’s correlation coefficients. These preliminary analyses were performed to identify participant characteristics associated with SWB and to inform the selection of variables for the subsequent adjusted analyses and multivariable modeling.

Separate one-way analyses of covariance (ANCOVA) were conducted to examine the associations between the weekly practice frequency of each individual yoga component (asana, pranayama, relaxation, and meditation) and SWB while adjusting for relevant participant and yoga practice characteristics. Each component-specific ANCOVA included place of residence, participation in other forms of physical exercise, yoga practice duration, and yoga expertise (yoga practitioner vs. yoga instructor) as covariates. Yoga practice duration and yoga expertise were additionally considered because they may be related to current practice frequency and could therefore represent relevant yoga-related characteristics when examining the associations between individual component frequency and SWB; yoga expertise was also significantly associated with SWB in the preliminary analyses (Table 1). Bonferroni-adjusted *post hoc* comparisons were conducted to examine pairwise differences between the four weekly practice frequency categories (once a week, 2–3 times a week, 4–5 times a week, and daily). To account for multiple testing across the four omnibus ANCOVA tests, the corresponding *p*-values were adjusted using the Holm–Bonferroni procedure.

**Table 1:** Associations between participant characteristics and subjective wellbeing (*N* = 379).

Variable	Statistical test	Test statistic	*p*	Effect size
Categorical variables				
Sex	One-way ANOVA	*F*(1, 377) = 0.566	0.452	ηp^2^ = 0.002
County	One-way ANOVA	*F*(19, 359) = 0.669	0.849	ηp^2^ = 0.034
Place of residence	One-way ANOVA	*F*(4, 374) = 2.736	0.029	ηp^2^ = 0.028
Educational attainment	One-way ANOVA	*F*(2, 376) = 0.202	0.817	ηp^2^ = 0.001
Occupation	One-way ANOVA	*F*(6, 372) = 1.808	0.096	ηp^2^ = 0.028
Yoga expertise	One-way ANOVA	*F*(1, 377) = 10.218	0.002	ηp^2^ = 0.026
Other physical exercise	One-way ANOVA	*F*(1, 377) = 4.798	0.029	ηp^2^ = 0.013
Continuous variables				
Age	Pearson correlation	*r* = 0.063	0.222	—
Yoga practice duration	Pearson correlation	*r* = 0.139	0.007	—

Subjective wellbeing was assessed using the WHO-5 Wellbeing Index. Categorical variables were analyzed using one-way ANOVA, and continuous variables were analyzed using Pearson’s correlation coefficients. Effect sizes for ANOVA are reported as partial eta squared (ηp^2^) and were interpreted according to conventional benchmarks (0.01 = small, 0.06 = medium, and 0.14 = large effects). For Pearson’s correlation coefficients, conventional benchmarks were applied (*r* = 0.10, small; *r* = 0.30, medium; *r* = 0.50, large).

A multiple linear regression analysis was subsequently performed to examine whether the weekly practice frequencies of the four individual yoga components were independently associated with SWB when considered simultaneously together with relevant participant and yoga practice characteristics. The predictors included place of residence, participation in other forms of physical exercise, yoga practice duration, yoga expertise, and the weekly practice frequencies of asana, pranayama, relaxation, and meditation. The four weekly practice-frequency variables were entered as ordinal predictors reflecting increasing frequency of practice (1 = once a week, 2 = 2–3 times a week, 3 = 4–5 times a week, 4 = daily). These codes were treated as approximately interval-scaled for three reasons. First, the categories are ordered and represent steps of broadly comparable size in weekly practice (roughly 1, 2.5, 4.5, and 7 sessions per week), although the steps are not strictly equal. Second, our hypotheses concerned a graded association between practice frequency and SWB, which a single linear term per component tests directly. Third, entering each component as one predictor rather than three dummy variables kept the model parsimonious (eight rather than 16 predictors) and consistent with the *a priori* power calculation. Place of residence was likewise entered as a five-level ordinal variable reflecting increasing settlement size, whereas yoga expertise and participation in other forms of physical exercise were dichotomous (coding is given in the note to Table 2). Spearman’s ρ correlations were also calculated to examine the associations among the weekly practice frequencies of the four yoga components (Supplementary Table S1).

**Table 2:** Multiple linear regression predicting subjective wellbeing (*N* = 379).

Predictor	*B*	SE	*β*	*t*	*p*	95% CI for *B*
Constant	6.58	0.85	—	7.72	<0.001	[4.90, 8.25]
Place of residence	0.15	0.12	0.06	1.23	0.220	[−0.09, 0.38]
Yoga practice duration	−0.001	0.03	−0.001	−0.02	0.984	[−0.06, 0.06]
Yoga expertise	0.24	0.36	0.04	0.68	0.500	[−0.46, 0.94]
Other physical exercise	−0.72	0.33	−0.11	−2.17	0.031	[−1.37, −0.07]
Asana frequency	0.69	0.24	0.19	2.87	0.004	[0.22, 1.17]
Pranayama frequency	0.25	0.22	0.08	1.13	0.260	[−0.18, 0.68]
Relaxation frequency	−0.31	0.24	−0.10	−1.32	0.188	[−0.78, 0.15]
Meditation frequency	0.61	0.20	0.20	3.01	0.003	[0.21, 1.00]

Dependent variable: subjective wellbeing (WHO-5). The regression model included participant characteristics (place of residence, yoga practice duration, yoga expertise, and participation in other forms of physical exercise) together with the weekly practice frequencies of the four individual yoga components. Categorical variables were coded as follows: place of residence (1 = village, 2 = small town, 3 = city [10,000–100,000 inhabitants], 4 = large city [over 100,000 inhabitants], 5 = capital city [Budapest]); yoga expertise (1 = yoga practitioner, 2 = yoga instructor); and other physical exercise besides yoga (1 = yes, 2 = no). The weekly practice frequency of each yoga component was coded 1 = once a week, 2 = 2–3 times a week, 3 = 4–5 times a week, and 4 = daily; yoga practice duration was entered in years. Place of residence and the four practice-frequency variables were entered as ordinal predictors. Overall model: *F*(8, 370) = 6.84, *p* < 0.001, *R*^2^ = 0.129, adjusted *R*^2^ = 0.110, Cohen’s *f*^2^ = 0.15 (medium effect), Durbin–Watson = 2.04. No evidence of problematic multicollinearity was observed (all VIFs < 2.3).

Because this linear treatment rests on an assumption of approximately equal spacing, its robustness was examined in a sensitivity analysis using categorical/dummy coding of the weekly practice-frequency variables. For each yoga component, the once-a-week category was used as the reference category, and three dummy variables were entered representing 2–3 times a week, 4–5 times a week, and daily practice. The sensitivity model therefore included 12 dummy variables for the four yoga components, together with the same four participant and yoga practice characteristics as the primary regression model, resulting in 16 predictors. The results of the sensitivity analysis were compared with those of the primary ordinally coded regression model to assess the robustness of the findings to the coding of the practice-frequency variables. Because the *a priori* sample-size calculation referred to the primary eight-predictor model (a 16-predictor model would require 420 participants under the same assumptions; achieved power with N = 379 ≈ 0.92) and the 12 category-specific coefficients were not adjusted for multiple testing, the sensitivity analysis was treated as secondary and exploratory.

Effect sizes were reported throughout. Partial eta squared (ηp^2^) was calculated for the ANOVA and ANCOVA models and interpreted using conventional benchmarks (small = 0.01, medium = 0.06, large = 0.14) (Tomczak and Tomczak, 2014). Pearson’s correlation coefficients were interpreted according to Cohen's (1988) guidelines (small = 0.10, medium = 0.30, large = 0.50). Cohen’s *d* was calculated for Bonferroni-adjusted pairwise comparisons and interpreted as small (0.20), medium (0.50), and large (0.80) effects (Fritz et al., 2012; Ialongo, 2016). The overall effect size of the multiple linear regression model was quantified using Cohen’s *f*^2^ and interpreted according to Cohen’s recommendations (small = 0.02, medium = 0.15, large = 0.35) (Cohen, 1988). No missing data were observed for any study variable. Statistical analyses were performed using IBM SPSS Statistics for macOS (Version 31.0; IBM Corp., Armonk, NY, USA). All statistical tests were two-tailed, and statistical significance was set at *p* < 0.05.

## 3 Results

### 3.1 Participant characteristics

A total of 379 Hungarian yoga practitioners completed the online survey. The sample was predominantly female (92.9%), with a mean age of 44.31 years (SD = 10.12; range = 20–75 years). Most participants had attained a bachelor’s degree or higher (75.2%), while 24.3% had completed secondary education and 0.5% primary education. Participants represented all counties of Hungary, with approximately one-third residing in Budapest (31.9%) and another one-third in medium-sized cities (31.4%) (Table 3).

**Table 3:** Sociodemographic and yoga practice characteristics of the participants (*N* = 379).

Age (years)	Mean ± SD (min-max) 44.31 ± 10.12 (20–75)	
Yoga practice duration (years)	Mean ± SD (min-max) 8.18 ± 6.36 (0.25–31)	
Characteristic	*n*	*%*
Sex		
Male	27	7.1
Female	352	92.9
Educational attainment		
Primary education	2	0.5
Secondary education	92	24.3
Bachelor’s degree or higher	285	75.2
Place of residence		
Village	53	14.0
Small town	37	9.8
City (10,000–100,000 inhabitants)	119	31.4
Large city (over 100,000 inhabitants)	49	12.9
Capital city (Budapest)	121	31.9
Occupational status		
Student	7	1.8
Self-employed	109	28.8
Casual employee	3	0.8
Unemployed	12	3.2
Employee / civil servant	205	54.1
Other	43	11.3
Yoga expertise		
Yoga practitioner	183	48.3
Yoga instructor	196	51.7
Other physical exercise (besides yoga)		
Yes	233	61.5
No	146	38.5

Values are presented as mean ± standard deviation (range) or frequency (%).

The mean duration of yoga practice was 8.18 years (SD = 6.36; range = 0.25–31 years). Slightly more than half of the participants were certified yoga instructors (51.7%), whereas 48.3% identified themselves as yoga practitioners. In addition to yoga, 61.5% reported engaging in other forms of physical exercise. Most participants were employed as employees or civil servants (54.1%) or were self-employed (28.8%) (Table 3).

All participants reported practicing each of the four investigated yoga components (i.e., asana, pranayama, relaxation, and meditation), although their weekly practice frequency differed across components. As shown in Table 4, asana, pranayama, and relaxation were most commonly practiced 2–3 times per week, whereas meditation was most frequently practiced once per week.

**Table 4:** Weekly practice frequency of the individual yoga components (N = 379).

Frequency	Asana		Pranayama		Relaxation		Meditation	
	*n*	%	*n*	%	*n*	%	*n*	%
Once a week	46	12.1	101	26.6	83	21.9	160	42.2
2–3 × a week	171	45.1	159	42.0	161	42.5	119	31.4
4–5 × a week	93	24.5	50	13.2	63	16.6	38	10.0
Daily	69	18.2	69	18.2	72	19.0	62	16.4
Total	379	100	379	100	379	100	379	100

*n* = frequency; % = percentage. Asana = physical postures; Pranayama = breathing techniques.

### 3.2 Associations between participant characteristics and subjective wellbeing

The assumptions underlying the statistical analyses were satisfied. For the categorical variables, the assumptions of one-way ANOVA were met, with normality supported by skewness and kurtosis values and homogeneity of variances confirmed using Levene’s test. Associations between categorical participant characteristics and SWB were examined using one-way ANOVA, whereas associations with continuous variables were assessed using Pearson’s correlation coefficients (Table 1).

Among the categorical participant characteristics, yoga expertise, participation in other forms of physical exercise, and place of residence were significantly associated with SWB. Yoga instructors reported higher SWB than yoga practitioners, *F*(1,377) = 10.22, *p* = 0.002, corresponding to a small effect (ηp^2^ = 0.026). Likewise, participants who engaged in other forms of physical exercise in addition to yoga reported higher SWB than those practicing yoga exclusively, *F*(1,377) = 4.80, *p* = 0.029, also representing a small effect (ηp^2^ = 0.013). A significant association was also observed between place of residence and SWB, *F*(4,374) = 2.74, *p* = 0.029, with a small effect size (ηp^2^ = 0.028). Participants living in villages and small towns generally reported higher SWB than those residing in larger urban areas (Table 1).

Pearson’s correlation analyses showed that yoga practice duration (in years) was weakly but significantly positively correlated with SWB (*r* = 0.139, *p* = 0.007), whereas age was not significantly correlated with SWB (*r* = 0.063, *p* = 0.222). Although the association between yoga practice duration and SWB reached statistical significance, its effect size was small, indicating limited practical significance.

Overall, the first hypothesis was partially supported. Selected participant characteristics, including place of residence, yoga expertise, participation in other forms of physical exercise, and, to a lesser extent, yoga practice duration, were associated with SWB, whereas the remaining sociodemographic characteristics showed no significant relationships. All four characteristics that were significantly associated with SWB (place of residence, participation in other forms of physical exercise, yoga practice duration, and yoga expertise) were subsequently accounted for in the adjusted analyses examining the associations between the weekly practice frequency of the individual yoga components and SWB.

### 3.3 Associations between weekly practice frequency of the individual yoga components and subjective wellbeing

The assumptions underlying the one-way analysis of covariance (ANCOVA) were satisfied. Normality was supported based on skewness and kurtosis values, linearity and homogeneity of regression slopes were confirmed by visual inspection and interaction tests, and Levene’s tests indicated homogeneity of variances across all analyses. After adjustment for place of residence, participation in other forms of physical exercise, yoga practice duration, and yoga expertise, weekly practice frequency was significantly associated with SWB for each of the four individual yoga components (Table 5). Separate ANCOVA models were conducted for asana, pranayama, relaxation, and meditation frequency, and all four omnibus associations remained statistically significant after Holm–Bonferroni correction. The associations were strongest for asana (ηp^2^ = 0.070) and meditation (ηp^2^ = 0.061), followed by pranayama (ηp^2^ = 0.050) and relaxation (ηp^2^ = 0.024). Adjusted means and 95% confidence intervals for each frequency category are presented in Supplementary Table S2.

**Table 5:** Adjusted associations between weekly practice frequency of individual yoga components and subjective wellbeing (one-way ANCOVA) (*N* = 379).

Variable	df	*F*	*p*	ηp^2^
Asana frequency	3	9.270	<0.001	0.070
Pranayama frequency	3	6.517	<0.001	0.050
Relaxation frequency	3	3.036	0.029	0.024
Meditation frequency	3	8.000	<0.001	0.061

Subjective wellbeing was assessed using the WHO-5 Wellbeing Index. Separate ANCOVA models were conducted for each yoga component. Covariates included place of residence, participation in other forms of physical exercise, yoga practice duration, and yoga expertise (yoga practitioner vs. yoga instructor). All four omnibus tests remained statistically significant after Holm–Bonferroni correction. Partial eta squared (ηp^2^) values indicate small (0.01), medium (0.06), and large (0.14) effect sizes.

For asana, Bonferroni-adjusted comparisons revealed a graded pattern across the frequency categories (Figure 1). Participants practicing asana more than once per week had significantly higher adjusted SWB than those practicing once weekly (2–3 times per week: mean difference = 1.756, 95% CI [0.333, 3.178], Cohen’s *d* = 0.52; 4–5 times per week: 1.941 [0.361, 3.520], *d* = 0.62; daily: 3.404 [1.683, 5.125], *d* = 1.08). Daily practitioners also reported significantly higher adjusted SWB than those practicing 2–3 times per week (mean difference = 1.648, 95% CI [0.395, 2.902], *d* = 0.52) and 4–5 times per week (1.463 [0.114, 2.813], *d* = 0.46), whereas the difference between the 2–3 and 4–5 times per week groups was not significant.

**Figure 1:**
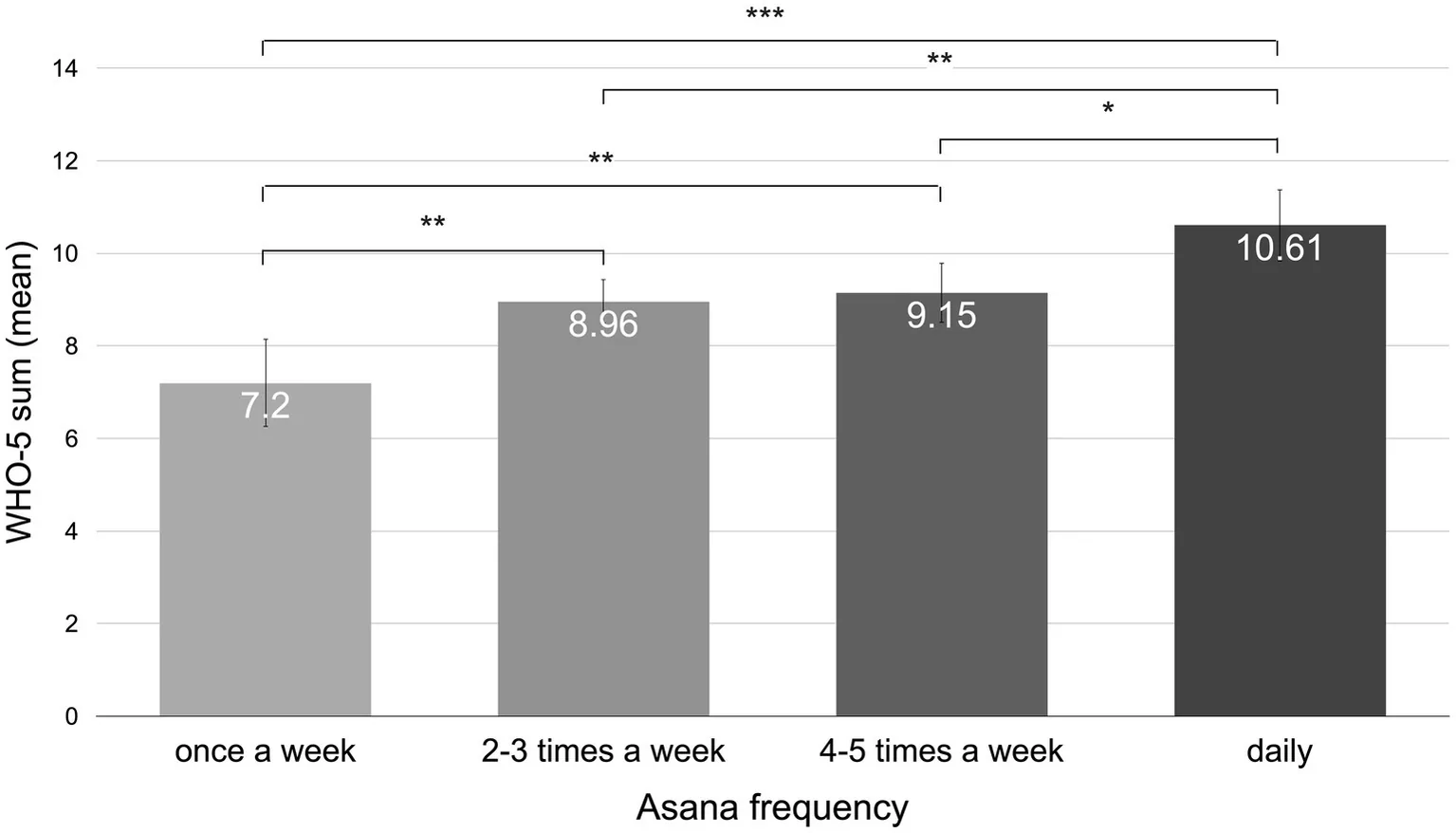
Mean subjective wellbeing (WHO-5) scores across weekly asana practice frequency groups. Adjusted means are presented after controlling for place of residence, participation in other forms of physical exercise, yoga practice duration, and yoga expertise. Error bars represent 95% confidence intervals. Pairwise group differences are based on Bonferroni-adjusted *post hoc* comparisons. *p < 0.05, **p < 0.01, ***p < 0.001.

A similar pattern was observed for pranayama (Figure 2). Participants practicing pranayama once weekly had significantly lower adjusted SWB than those practicing 2–3 times per week (mean difference = 1.238, 95% CI [0.153, 2.323], Cohen’s *d* = 0.40), 4–5 times per week (1.764 [0.259, 3.269], *d* = 0.56), or daily (2.152 [0.752, 3.551], *d* = 0.73). No significant differences were observed among the three higher-frequency groups.

**Figure 2:**
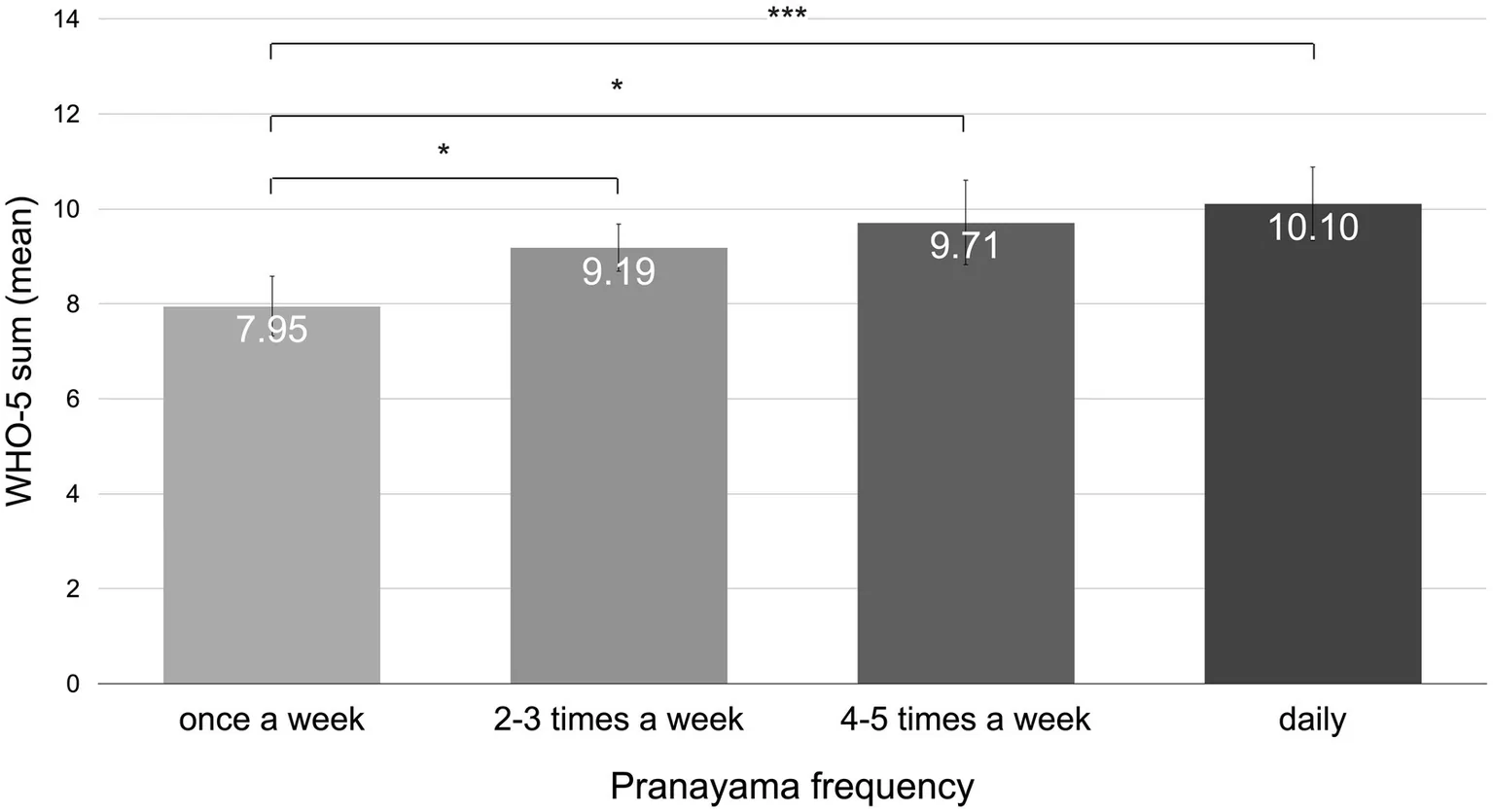
Mean subjective wellbeing (WHO-5) scores across weekly pranayama practice frequency groups. Adjusted means are presented after controlling for place of residence, participation in other forms of physical exercise, yoga practice duration, and yoga expertise. Error bars represent 95% confidence intervals. Pairwise group differences are based on Bonferroni-adjusted *post hoc* comparisons. *p < 0.05, ***p < 0.001.

For relaxation, the only significant pairwise difference was between once-weekly and daily practice (Figure 3), with daily practitioners showing higher adjusted SWB (mean difference = 1.688, 95% CI [0.194, 3.182], Cohen’s *d* = 0.60). No other pairwise comparisons were significant.

**Figure 3:**
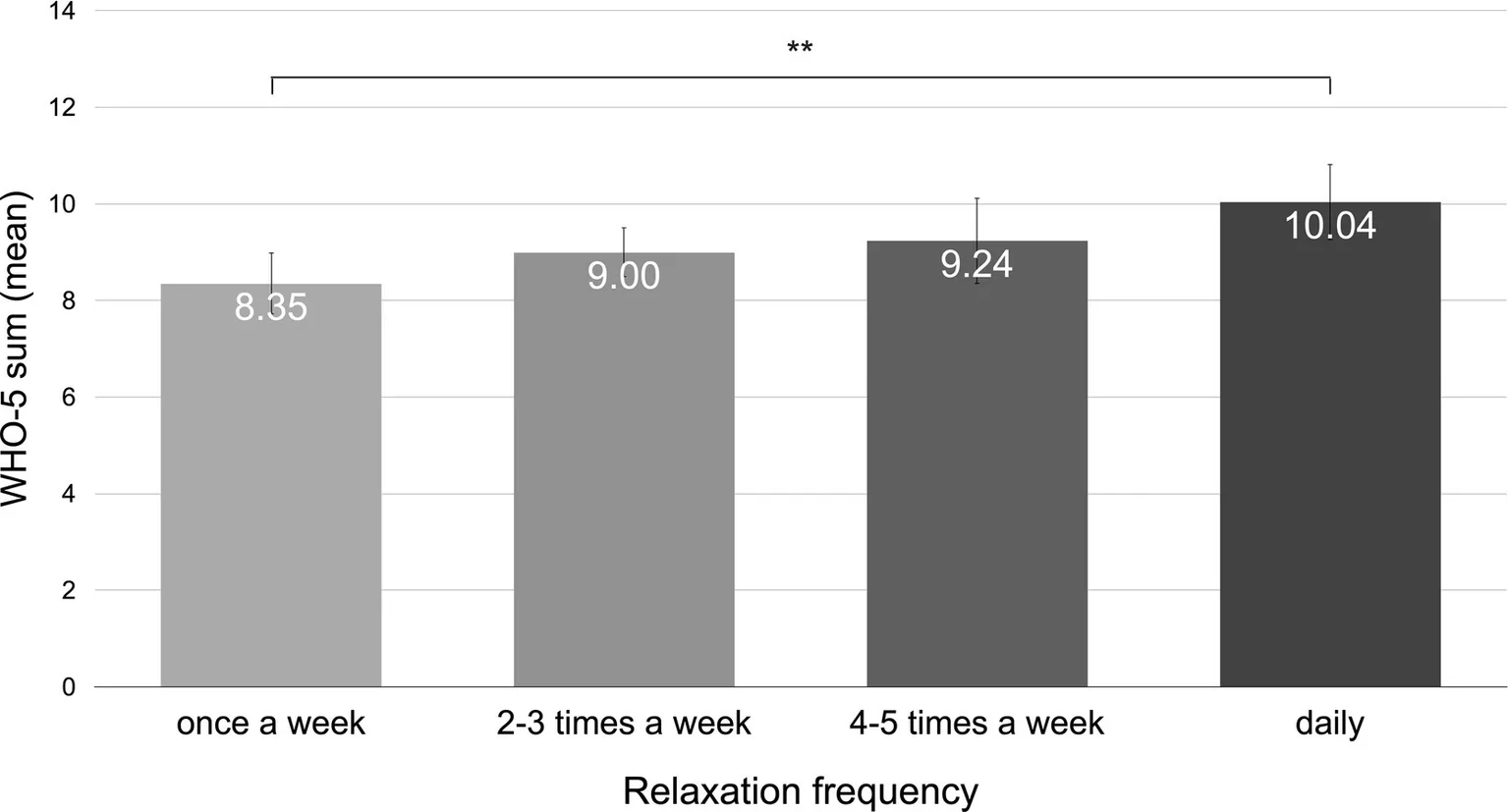
Mean subjective wellbeing (WHO-5) scores across weekly relaxation practice frequency groups. Adjusted means are presented after controlling for place of residence, participation in other forms of physical exercise, yoga practice duration, and yoga expertise. Error bars represent 95% confidence intervals. Pairwise group differences are based on Bonferroni-adjusted *post hoc* comparisons. **p < 0.01.

For meditation, participants practicing 2–3 times per week and daily had significantly higher adjusted SWB than those practicing once weekly (Figure 4), with mean differences of 1.241 (95% CI [0.212, 2.269], Cohen’s *d* = 0.40) and 2.397 (95% CI [1.010, 3.783], *d* = 0.83), respectively. No other pairwise comparisons were significant.

**Figure 4:**
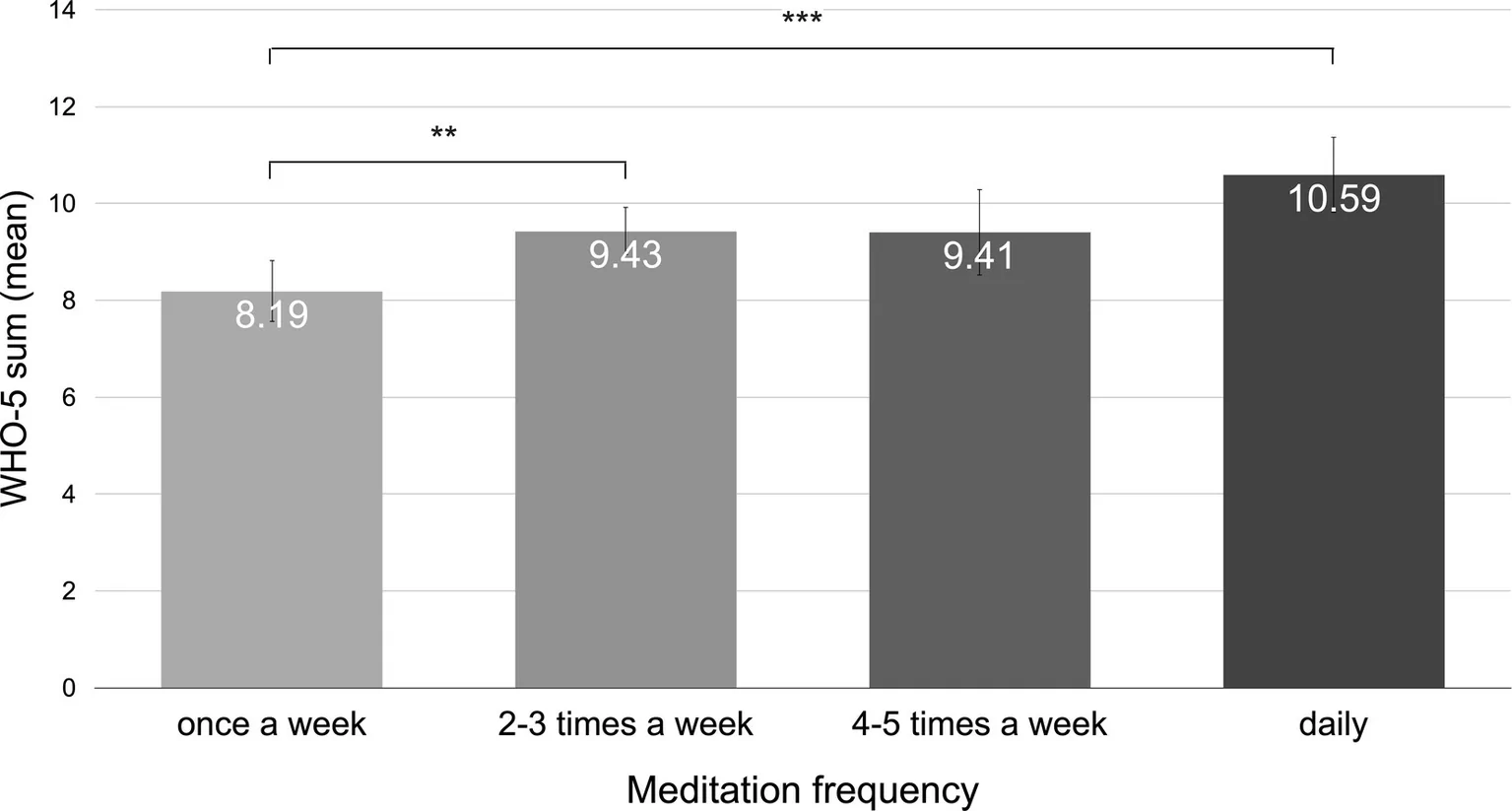
Mean subjective wellbeing (WHO-5) scores across weekly meditation practice frequency groups. Adjusted means are presented after controlling for place of residence, participation in other forms of physical exercise, yoga practice duration, and yoga expertise. Error bars represent 95% confidence intervals. Pairwise group differences are based on Bonferroni-adjusted *post hoc* comparisons. **p < 0.01, ***p < 0.001.

Overall, the second hypothesis was supported. More frequent practice of each individual yoga component was associated with higher SWB after adjustment for the significant participant and yoga practice characteristics identified in the preliminary analyses. To determine whether these associations remained independent when all significant variables were considered simultaneously, a multiple linear regression analysis was subsequently performed.

### 3.4 Independent correlates of subjective wellbeing

To identify the variables independently associated with SWB, a multiple linear regression analysis (Enter method) was conducted (Table 2). The regression model included the participant characteristics identified in the preliminary analyses (place of residence, yoga practice duration, yoga expertise, and participation in other forms of physical exercise) together with the weekly practice frequencies of the four individual yoga components. The overall regression model was statistically significant, *F*(8, 370) = 6.84, *p* < 0.001, explaining 12.9% of the variance in SWB (*R*^2^ = 0.129, adjusted *R*^2^ = 0.110), corresponding to an overall effect size at the threshold between small and medium (Cohen’s *f*^2^ = 0.15). In other words, roughly 87% of the variance in SWB was not accounted for by the variables in the model.

Diagnostic analyses confirmed that the assumptions of multiple linear regression were satisfied. The Durbin–Watson statistic (2.04) indicated independence of residuals, standardized residuals revealed no substantial outliers, and collinearity diagnostics indicated no evidence of problematic multicollinearity (all VIFs < 2.3). The weekly practice frequencies of the four yoga components were positively correlated with one another (Supplementary Table S1), with Spearman’s ρ values ranging from 0.470 to 0.631; however, these associations did not result in problematic multicollinearity in the regression model.

Among the variables included in the model, weekly asana practice frequency was independently associated with higher SWB (*B* = 0.69, *β* = 0.19, 95% CI [0.22, 1.17], *p* = 0.004), as was weekly meditation practice frequency (*B* = 0.61, *β* = 0.20, 95% CI [0.21, 1.00], *p* = 0.003). Participation in other forms of physical exercise also remained significantly associated with SWB (*B* = −0.72, *β* = −0.11, 95% CI [−1.37, −0.07], *p* = 0.031); because this variable was coded 1 = yes and 2 = no, the negative coefficient indicates lower SWB among participants who did not engage in other forms of exercise. In contrast, the confidence intervals for place of residence (*B* = 0.15, 95% CI [−0.09, 0.38]), yoga practice duration (*B* = −0.001, 95% CI [−0.06, 0.06]), yoga expertise (*B* = 0.24, 95% CI [−0.46, 0.94]), pranayama frequency (*B* = 0.25, 95% CI [−0.18, 0.68]), and relaxation frequency (*B* = −0.31, 95% CI [−0.78, 0.15]) all included zero, indicating no statistically significant independent associations with SWB in the model.

### 3.5 Sensitivity analysis using categorical coding

To examine whether the findings were sensitive to the treatment of weekly practice frequency as an ordinal predictor, a sensitivity multiple linear regression analysis was conducted using categorical/dummy coding, with once-a-week practice as the reference category for each yoga component. The model included 16 predictors: 12 dummy variables representing the three higher-frequency categories of each of the four yoga components, together with place of residence, other physical exercise, yoga practice duration, and yoga expertise. The overall model was statistically significant, *F*(16, 362) = 4.240, *p* < 0.001, *R*^2^ = 0.158, adjusted *R*^2^ = 0.121, corresponding to a medium effect size (Cohen’s *f*^2^ = 0.19). No evidence of problematic multicollinearity was observed, with the maximum VIF being 3.655 (Supplementary Table S3).

The categorical analysis showed that all three higher-frequency asana categories were significantly associated with higher SWB compared with once-weekly practice: 2–3 times/week (*B* = 1.560, 95% CI [0.390, 2.730], *p* = 0.009), 4–5 times/week (*B* = 1.428, 95% CI [0.073, 2.784], *p* = 0.039), and daily practice (*B* = 3.022, 95% CI [1.465, 4.578], *p* < 0.001). For meditation, practicing 2–3 times/week (*B* = 1.245, 95% CI [0.391, 2.098], *p* = 0.004) and daily (*B* = 1.807, 95% CI [0.550, 3.064], *p* = 0.005) were significantly associated with higher SWB compared with once-weekly practice, whereas the 4–5 times/week category was not statistically significant (*B* = 0.662, 95% CI [−0.662, 1.986], *p* = 0.326). None of the relaxation-frequency categories differed significantly from once-weekly practice: 2–3 times/week (*B* = −0.654, 95% CI [−1.672, 0.365], *p* = 0.208), 4–5 times/week (*B* = −0.581, 95% CI [−1.884, 0.721], *p* = 0.381), or daily practice (*B* = −0.837, 95% CI [−2.315, 0.641], *p* = 0.266).

For pranayama, the 4–5 times/week category was significantly associated with higher SWB compared with once-weekly practice (*B* = 1.437, 95% CI [0.146, 2.727], *p* = 0.029), whereas the 2–3 times/week (*B* = 0.572, 95% CI [−0.342, 1.487], *p* = 0.219) and daily (*B* = 0.453, 95% CI [−0.945, 1.850], *p* = 0.525) categories were not statistically significant. Thus, the categorical sensitivity analysis broadly supported the main pattern of the ordinally coded model for asana and meditation, while revealing category-specific differences for pranayama and showing no statistically significant category-specific associations for relaxation (Supplementary Table S3).

Overall, the categorical sensitivity analysis was broadly consistent with the primary ordinally coded model, particularly for asana and meditation, while revealing some category-specific differences for pranayama and relaxation. Yoga practice duration was not independently associated with SWB in the categorical model (*B* = 0.004, 95% CI [−0.055, 0.063], *p* = 0.897). These findings support the robustness of the main associations while indicating some variation across specific practice-frequency categories.

## 4 Discussion

The present study examined whether the weekly practice frequency of individual yoga components was associated with SWB among Hungarian yoga practitioners after accounting for relevant sociodemographic and lifestyle characteristics. Overall, the findings supported the second hypothesis and partially supported the first and third hypotheses. First, place of residence, yoga expertise, participation in other forms of physical exercise, and yoga practice duration were significantly associated with SWB, although all of these associations were small. Second, higher weekly practice frequency of each individual yoga component (asana, pranayama, relaxation, and meditation) was associated with higher SWB after adjustment for the identified covariates, with medium effect sizes observed for asana and meditation, and small effect sizes for pranayama and relaxation. Finally, after simultaneously accounting for participant characteristics, yoga experience variables, and the overlap among the individual yoga components, only the weekly practice frequencies of asana and meditation remained independently and positively associated with SWB, whereas yoga practice duration, yoga expertise, and the remaining yoga components did not explain additional variance in the outcome. These associations were modest, however. The full model accounted for only 12.9% of the variance in SWB, so the findings point to small independent associations of current asana and meditation practice with SWB rather than to major determinants of wellbeing, and, given the cross-sectional design, they cannot tell us in which direction these associations run.

### 4.1 Yoga practitioner characteristics and SWB

Among the seven categorical participant characteristics examined, only three showed statistically significant associations with SWB, whereas the remaining categorical variables were not associated with the outcome. In addition, Pearson’s correlation analyses indicated a weak but statistically significant positive association between yoga practice duration and SWB (*r* = 0.139), whereas age was not significantly correlated with SWB. Specifically, participants living in villages or small towns, yoga instructors, and those engaging in other forms of physical exercise in addition to yoga reported significantly higher SWB than their respective comparison groups. All of these associations were small. In contrast, sex, age, county of residence, educational attainment, and occupational status were not significantly associated with SWB. Collectively, these findings suggest that although selected participant characteristics may be related to individual differences in SWB, their practical importance appears limited.

The present finding that yoga practitioners living in villages and small towns reported higher SWB than those residing in larger urban areas is generally consistent with previous research suggesting that urban residence is often associated with poorer mental health outcomes (Xu et al., 2023). However, the available evidence remains heterogeneous, and the association appears to be driven less by the urban–rural distinction itself than by contextual factors such as population density, neighborhood quality, and social cohesion (Xu et al., 2023). Therefore, the higher SWB observed among participants living in smaller settlements may reflect more favorable psychosocial and environmental conditions. Nevertheless, given the small effect size, this finding should be interpreted with caution.

Participants who engaged in other forms of physical exercise in addition to yoga also reported significantly higher SWB than those practicing yoga alone. Direct comparison with previous research is challenging because most studies have compared yoga with other forms of physical activity rather than examining the potential added benefit of combining yoga with additional exercise. A meta-analysis found that yoga improved psychological wellbeing compared with no intervention but showed no advantage over other forms of physical activity (Hendriks et al., 2017), while observational studies suggest that higher wellbeing among yoga practitioners is primarily associated with regular yoga practice itself (Cartwright et al., 2020; Cseh et al., 2026). Therefore, the present finding may reflect the generally higher wellbeing of people maintaining an overall physically active lifestyle rather than an association specific to combining yoga with other forms of exercise.

Yoga instructors also reported significantly higher SWB than non-teaching yoga practitioners, although the observed effect size was small. Direct comparisons between yoga instructors and non-teaching practitioners are currently lacking. Nevertheless, previous studies consistently suggest that long-term yoga practitioners report better mental health than non-practitioners (Campelo et al., 2025; Gothe et al., 2019), while national surveys from Germany and the United Kingdom have demonstrated high levels of wellbeing and lower perceived stress among yoga practitioners (Cartwright et al., 2020; Cramer et al., 2019). Therefore, yoga instructor status may reflect greater long-term engagement with yoga practice rather than an independent correlate of SWB. However, given the limited available evidence and the small effect size observed, this finding should be interpreted with caution.

A weak positive association was also observed between yoga practice duration and SWB, although the effect size was small. This finding is consistent with that of Ross et al. (2013), suggesting that accumulated years of yoga practice may be related to wellbeing. In contrast, national surveys conducted in Germany and the United Kingdom did not identify a significant association between yoga practice duration and wellbeing, whereas the importance of regular weekly practice was consistently demonstrated across studies (Cartwright et al., 2020; Cramer et al., 2019). Together, these findings suggest that yoga practice duration may represent a relevant, albeit modest, correlate of SWB, whereas the psychological benefits of regular weekly yoga practice appear to be more consistently supported by the available evidence.

These findings partially support the first hypothesis, indicating that selected participant characteristics are associated with SWB, although their practical relevance appears modest compared with the weekly practice frequency of the individual yoga components.

### 4.2 Weekly practice frequency of individual yoga components and SWB

After adjustment for the identified participant and yoga practice characteristics, higher weekly practice frequency of all four yoga components was significantly associated with higher SWB. However, medium effect sizes were observed only for asana (ηp^2^ = 0.070) and meditation (ηp^2^ = 0.061), whereas pranayama (ηp^2^ = 0.050) and relaxation (ηp^2^ = 0.024) demonstrated small effect sizes. Even for asana, weekly practice frequency accounted for only about 7% of the variance in SWB after adjustment. These findings are broadly consistent with current evidence indicating that the individual yoga components are not equivalent in their associations with mental health and wellbeing, although the available evidence remains heterogeneous because most interventions combine several yoga practices rather than isolate their individual effects (Campelo et al., 2025; Frazier et al., 2026). Comparative studies likewise suggest that breathing exercises, meditation, and multicomponent yoga may contribute more strongly to psychological wellbeing and stress reduction than single-component approaches, while posture-based practice appears to play an important role in overall mental health (Kushwaha et al., 2024; Novaes et al., 2020). Nevertheless, the strongest evidence continues to support a comprehensive yoga practice integrating multiple components rather than emphasizing any single practice in isolation.

The findings further suggest that more frequent weekly practice of individual yoga components is associated with progressively higher SWB, with the highest scores generally observed among participants practicing daily. This pattern is consistent with previous observational studies reporting a positive association between the weekly practice frequency of yoga and SWB assessed using the WHO-5 (Tornóczky et al., 2026), as well as between the frequency of individual yoga components and SWB (Cseh et al., 2026). Review-level evidence likewise supports a dose–response relationship between yoga practice frequency and mental health, with higher weekly practice frequency being associated with greater reductions in depressive symptoms, although evidence for positive wellbeing outcomes remains less consistent (Brinsley et al., 2021). If these associations were at least partly causal, they could reflect cumulative psychological and physiological effects of regular practice, including enhanced stress regulation, mindfulness, and emotion regulation (Pascoe et al., 2021). Regular yoga practice may additionally enhance wellbeing through improved autonomic regulation, greater vagal activity, and enhanced neuroplasticity, providing plausible biological mechanisms for the observed associations (Upadhyay et al., 2023). However, these mechanisms were not assessed in the present cross-sectional study, and the reverse direction, namely that individuals with higher SWB practice more frequently, is equally plausible. The pandemic context adds a further layer of uncertainty. Data were collected in spring 2021, when daily routines, access to group classes, and wellbeing were all affected by pandemic-related restrictions. Studies conducted during the pandemic have also reported better mental health among regular yoga practitioners (Malipeddi et al., 2024; Upadhyay et al., 2022), but practitioners who managed to keep up frequent practice at that time may have differed from others in free time, living conditions, or psychological resources, all of which could be related to both practice frequency and SWB.

Overall, these findings support the second hypothesis and suggest that more frequent weekly engagement in all major yoga components is associated with higher SWB, although the magnitude of these associations differs across individual components.

### 4.3 Independent associations of individual yoga components with SWB

The multiple linear regression analysis represents the principal novel finding of the present study. The overall model showed an effect size at the small-to-medium threshold (Cohen’s *f*^2^ = 0.15), but explained only 12.9% of the variance in SWB (*R*^2^ = 0.129; adjusted *R*^2^ = 0.110), indicating that the variables included in the model accounted for a relatively modest proportion of individual differences in SWB. The independent associations of asana (*β* = 0.19) and meditation (*β* = 0.20) with higher SWB were small in magnitude, while participation in other forms of physical exercise also showed a small association (*β* = −0.11), reflecting the coding of this variable. The modest explained variance further suggests that other psychological, social, lifestyle, and contextual factors not assessed in the present study may account for a substantial proportion of the variability in SWB. Asana and meditation frequency should therefore not be regarded as major determinants of SWB; they are better understood as two of many factors that were modestly associated with SWB in this sample. After adjustment for participant characteristics, yoga experience variables, and the shared variance among the individual yoga components, current weekly engagement in asana and meditation was independently associated with higher SWB, whereas yoga practice duration, yoga expertise, pranayama, and relaxation were not. Although yoga practice duration showed a weak positive bivariate association with SWB, it was not independently associated with SWB in the multivariable model, suggesting that current practice frequency may be more closely associated with SWB than accumulated years of yoga practice.

One possible explanation for the independent associations of asana and meditation is that these practices may engage complementary physical and psychological processes, whereas pranayama and relaxation may share overlapping variance with these components, reducing their unique associations with SWB in the regression model (Matko et al., 2021).

To the best of our knowledge, only a few previous studies have examined the independent association of individual yoga components within the same multivariable model, making direct comparison difficult. Existing reviews consistently suggest that different yoga components contribute differently to mental health, while emphasizing that most interventions combine several practices, limiting conclusions regarding the unique association of individual components (Frazier et al., 2026; Matko et al., 2021; Mu et al., 2026). Similarly, simultaneous analyses have reported positive associations between all major yoga components and wellbeing without identifying clear independent predictors (Cseh et al., 2026). Thus, an important research gap remains regarding the independent associations of individual yoga components with wellbeing after accounting for their shared variance and relevant participant characteristics. The present findings therefore extend the current evidence by suggesting that, when the overlap among yoga components and yoga experience variables is statistically accounted for, asana and meditation show the strongest, though still small, independent associations with SWB among the four components.

Importantly, the sensitivity analysis using categorical/dummy coding of the weekly practice-frequency variables provided additional support for the main findings while also illustrating some category-specific differences that are not captured by ordinal coding. The associations of asana and meditation with SWB were supported by the categorical specification, whereas relaxation showed no significant category-specific associations. For pranayama, the categorical analysis identified a significant association for the 4–5 times/week category compared with once-weekly practice, although the overall ordinally coded predictor was not significant in the primary regression model. These findings indicate that the relationship between practice frequency and SWB may not be fully represented by a single linear trend across the four frequency categories. Accordingly, the results should be interpreted in terms of associations between specific frequency categories and SWB rather than as evidence of a uniform linear increase across all frequency levels. Because the 12 category-specific coefficients were not corrected for multiple testing and the 16-predictor model was not covered by the *a priori* sample-size calculation, the isolated pranayama finding in particular should be regarded as exploratory.

Accordingly, the third hypothesis was only partially supported, as not all individual yoga components remained independent predictors after adjustment for participant characteristics and the overlap among yoga components.

### 4.4 Strengths and limitations

The present study has several important strengths. To our knowledge, it is among the first studies to examine the independent association of the weekly practice frequency of individual yoga components with SWB within the same multivariable model. By moving beyond yoga as a single, homogeneous intervention, the study provides a more nuanced understanding of how different components may be differentially related to positive mental health. An additional strength is the assessment of SWB using the WHO-5, a widely used measure of positive mental health, together with the consistent reporting and interpretation of effect sizes, which facilitates evaluation of the practical significance of the findings.

Several limitations need to be kept firmly in mind when interpreting these findings, and the most important of them concern the study design and the way participants were recruited. Because the data are cross-sectional, the associations reported here cannot be interpreted as effects of yoga practice on SWB, and their direction is unknown. It is at least as plausible that people who feel better are more inclined, or simply more able, to practice frequently as it is that frequent practice raises SWB, and unmeasured third variables such as personality, physical health, free time, or social support could underlie both. Participants were also recruited through a convenience snowball procedure that relied on HYTA-affiliated instructors forwarding the invitation to their students and professional networks. This approach probably favored practitioners who were closely connected to organized yoga communities and motivated to take part, and it did not allow a response rate to be calculated. All participants practiced at least once a week, so the findings describe differences among regular practitioners and cannot be extended to occasional practitioners or to non-practitioners. More than half of the sample (51.7%) were yoga instructors, a proportion that almost certainly reflects the recruitment route rather than the composition of the Hungarian yoga community. For these reasons, the sample cannot be regarded as representative of Hungarian yoga practitioners, and the results should be generalized only with considerable caution.

The demographic composition of the sample restricts generalizability further. Nearly all respondents were women (92.9%), and three quarters held at least a bachelor’s degree. Women and men may differ in their reasons for practicing yoga, in the styles and components they prefer, and in how they report their wellbeing, and educational attainment is itself related to health behaviors and wellbeing. With only 27 men in the sample, sex-specific analyses were not feasible. The associations observed here may therefore be different, weaker, or absent among male practitioners and among practitioners with lower educational attainment, and they should not be assumed to hold for the wider yoga-practicing population.

Measurement is a further limitation. All variables were self-reported, and the weekly frequency of each component was assessed with a single item that depended on participants’ estimate of a typical week and on their own classification of what they practiced. This leaves room for two kinds of bias. The first is recall bias: estimating a typical week is imprecise, and some respondents may have overstated how often they practice, particularly those for whom yoga is central to their identity, such as instructors. The second is classification bias. Yoga sessions often blend components (for example, breath-synchronized posture sequences, breathing exercises used as a lead-in to meditation, or guided relaxation that could just as well be described as meditation), so the same activity may have been labeled differently by different respondents. The moderate-to-strong intercorrelations among the four frequency items (ρ = 0.47–0.63) are consistent with such overlap. If this misclassification was essentially random, it would most likely have weakened the component-specific associations; if it was systematic (for instance, if instructors classified their practice more precisely than other practitioners), it could have biased them in either direction. Moreover, the reliability and validity of these single items have not been established, and because exposure and outcome were reported by the same respondents at the same time, shared method variance may have inflated the observed associations.

The timing of data collection is an important contextual limitation as well. The survey ran from mid-April to mid-May 2021, as Hungary was coming out of a severe third wave of the COVID-19 pandemic and restrictions on leisure and sports facilities were being lifted only gradually. For many practitioners, group classes had moved online or had been replaced by home practice, and the pandemic itself affected population wellbeing. Both the practice frequencies and the SWB levels in our sample may therefore differ from those under ordinary conditions, and the associations between them may also have been shaped by the circumstances, for example if yoga served as a particularly important coping resource at the time, or if those able to maintain frequent practice had more time, space, or psychological resources than others. These influences could not be assessed directly, and the findings may not generalize to non-pandemic conditions.

Finally, the study did not examine other potentially relevant aspects of yoga practice, including yoga style, practice intensity, session duration, instructional quality, or potential psychological mediators. In addition, a substantial proportion of participants engaged in other forms of physical exercise besides yoga, which makes it difficult to disentangle the relative associations of yoga practice and overall physical activity with SWB. Future longitudinal and intervention studies should address these factors and recruit more diverse and representative samples, including more men and practitioners with lower educational attainment. Such studies would also benefit from validated, component-specific measures of practice, ideally supplemented by practice diaries or ecological momentary assessment to reduce recall and classification bias.

## 5 Conclusion

The present study suggests that more frequent weekly practice of all major yoga components is associated with higher SWB in this convenience sample of predominantly female, highly educated Hungarian yoga practitioners, with the strongest associations observed for asana and meditation, although all of these associations were modest. However, after accounting for participant characteristics, yoga experience variables, and overlap among individual yoga components, in the primary ordinally coded regression model, only asana and meditation remained independently associated with higher SWB, and the model as a whole accounted for only 12.9% of its variance. Furthermore, the findings are consistent with the view that yoga may be better understood as a multidimensional mind–body practice rather than a homogeneous one, as its individual components showed distinct patterns of association with SWB.

Because the design was cross-sectional, the sample was a convenience sample of mostly highly educated women, and the data were collected during the COVID-19 pandemic, causal conclusions cannot be drawn and the findings should be generalized with caution. Within these limits, the findings highlight the relevance of considering both the weekly practice frequency and specific yoga components in future research investigating the relationship between yoga practice and positive mental health. Future longitudinal and intervention studies should further examine the unique and interactive associations of individual yoga components with SWB and determine whether these relationships are replicated across different populations and study designs.

## Data Availability

The original contributions presented in the study are included in the article/Supplementary material, further inquiries can be directed to the corresponding author/s.
