# Supplementary material for: Weekly practice frequency of individual yoga components and subjective wellbeing in Hungarian yoga practitioners: a nationwide cross-sectional online survey
Source: Front Psychol. 2026 Oct 28;17:1941678. doi: 10.3389/fpsyg.2026.1941678 (PMC13751533; doi:10.3389/fpsyg.2026.1941678)
Supplement: Supplementary file 1 [file Table_1.DOCX]

Supplementary Material

Weekly practice frequency of individual yoga components and subjective wellbeing in Hungarian yoga practitioners: a national cross-sectional study

Gusztáv József Tornóczky^1,2*^, Sunil Singh Yadav^3^, Henriett Nagy^4^, İsmail İlbak^5^, Vera Klier^6^, Dorottya Bosnyák^1,6^, Attila Szabo^7^, Réka Erika Kovács^7^

**Supplementary Tables**

**Table S1.** Descriptive statistics and Spearman's rho correlations among weekly practice frequencies of the four yoga components (N = 379)

| **Variable** | ***N*** | ***M*** | ***SD*** | **1** | **2** | **3** | **4** |
| --- | --- | --- | --- | --- | --- | --- | --- |
| 1. Asana frequency | 379 | 2.49 | 0.927 | — |  |  |  |
| 2. Pranayama frequency | 379 | 2.23 | 1.038 | 0.587*** | — |  |  |
| 3. Relaxation frequency | 379 | 2.33 | 1.020 | 0.597*** | 0.590*** | — |  |
| 4. Meditation frequency | 379 | 2.01 | 1.086 | 0.398*** | 0.507*** | 0.597*** | — |

Values below the diagonal represent Spearman's rho correlation coefficients. All correlations were statistically significant, *p* < 0.001 (two-tailed). The 95% confidence intervals were estimated using Fisher's *r*-to-*z* transformation, with standard errors estimated according to the formula proposed by Fieller, Hartley, and Pearson. The 95% confidence intervals were as follows: asana–pranayama [0.515, 0.651], asana–relaxation [0.526, 0.660], asana–meditation [0.307, 0.482], pranayama–relaxation [0.518, 0.654], pranayama–meditation [0.426, 0.580], and relaxation–meditation [0.526, 0.660]. ***p < 0.001, two-tailed.

**Table S2.** Adjusted means and 95% confidence intervals for subjective wellbeing by weekly practice frequency of individual yoga components (N = 379)

| **Yoga component** | **Weekly practice frequency** | **Adjusted mean** | **95% CI** |
| --- | --- | --- | --- |
| Asana | Once a week | 7.116 | 6.179–8.053 |
|  | 2–3 times a week | 8.947 | 8.470–9.425 |
|  | 4–5 times a week | 9.166 | 8.519–9.813 |
|  | Daily | 10.670 | 9.900–11.439 |
| Pranayama | Once a week | 7.949 | 7.311–8.587 |
|  | 2–3 times a week | 9.186 | 8.686–9.687 |
|  | 4–5 times a week | 9.713 | 8.816–10.610 |
|  | Daily | 10.100 | 9.315–10.886 |
| Relaxation | Once a week | 8.348 | 7.629–9.067 |
|  | 2–3 times a week | 8.998 | 8.493–9.503 |
|  | 4–5 times a week | 9.237 | 8.428–10.046 |
|  | Daily | 10.035 | 9.243–10.828 |
| Meditation | Once a week | 8.188 | 7.680–8.697 |
|  | 2–3 times a week | 9.429 | 8.853–10.005 |
|  | 4–5 times a week | 9.408 | 8.385–10.432 |
|  | Daily | 10.585 | 9.734–11.436 |

Adjusted means and 95% confidence intervals were estimated from separate one-way ANCOVA models for each yoga component, adjusting for place of residence, participation in other forms of physical exercise, yoga practice duration, and yoga expertise (yoga practitioner vs. yoga instructor). Covariates were evaluated at their observed mean values: place of residence = 2.61, other exercise = 1.39, yoga practice duration = 8.1834 years, and yoga expertise = 1.52. Subjective wellbeing was assessed using the WHO-5 Well-Being Index.

**Table S3.** Multiple linear regression using categorical coding of weekly yoga component practice frequency (N = 379)

| **Predictor** | **B** | **SE** | **β** | **t** | ***p*** | **95% CI for B** |
| --- | --- | --- | --- | --- | --- | --- |
| Constant | 7.19 | 0.88 | — | 8.21 | <0.001 | [5.47, 8.91] |
| Place of residence | 0.14 | 0.12 | 0.06 | 1.18 | 0.237 | [−0.09, 0.37] |
| Yoga practice duration | 0.004 | 0.03 | 0.007 | 0.13 | 0.897 | [−0.06, 0.06] |
| Yoga expertise | 0.13 | 0.39 | 0.02 | 0.33 | 0.744 | [−0.63, 0.89] |
| Other physical exercise | −0.74 | 0.33 | −0.11 | −2.22 | 0.027 | [−1.39, −0.08] |
| Asana 2–3 times/week | 1.56 | 0.60 | 0.23 | 2.62 | 0.009 | [0.39, 2.73] |
| Asana 4–5 times/week | 1.43 | 0.69 | 0.19 | 2.07 | 0.039 | [0.07, 2.78] |
| Asana daily | 3.02 | 0.79 | 0.35 | 3.82 | <0.001 | [1.47, 4.58] |
| Pranayama 2–3 times/week | 0.57 | 0.47 | 0.09 | 1.23 | 0.219 | [−0.34, 1.49] |
| Pranayama 4–5 times/week | 1.44 | 0.66 | 0.15 | 2.19 | 0.029 | [0.15, 2.73] |
| Pranayama daily | 0.45 | 0.71 | 0.05 | 0.64 | 0.525 | [−0.95, 1.85] |
| Relaxation 2–3 times/week | −0.65 | 0.52 | −0.10 | −1.26 | 0.208 | [−1.67, 0.37] |
| Relaxation 4–5 times/week | −0.58 | 0.66 | −0.07 | −0.88 | 0.381 | [−1.88, 0.72] |
| Relaxation daily | −0.84 | 0.75 | −0.10 | −1.11 | 0.266 | [−2.32, 0.64] |
| Meditation 2–3 times/week | 1.25 | 0.43 | 0.17 | 2.87 | 0.004 | [0.39, 2.10] |
| Meditation 4–5 times/week | 0.66 | 0.67 | 0.06 | 0.98 | 0.326 | [−0.66, 1.99] |
| Meditation daily | 1.81 | 0.64 | 0.20 | 2.83 | 0.005 | [0.55, 3.06] |

Dependent variable: subjective wellbeing (WHO-5). B = unstandardized regression coefficient; SE = standard error; β = standardized regression coefficient; CI = confidence interval. The regression model included place of residence, yoga practice duration, yoga expertise, participation in other forms of physical exercise, and the weekly practice frequencies of the four individual yoga components. For each yoga component, once-a-week practice was used as the reference category. Categorical variables were coded as follows: place of residence (1 = village, 2 = small town, 3 = city [10,000–100,000 inhabitants], 4 = large city [over 100,000 inhabitants], 5 = capital city [Budapest]); yoga expertise (1 = yoga practitioner, 2 = yoga instructor); and other physical exercise besides yoga (1 = yes, 2 = no). Yoga practice duration was entered in years. Overall model: *F*(16, 362) = 4.240, *p* < 0.001, *R*² = 0.158, adjusted *R*² = 0.121. No evidence of problematic multicollinearity was observed (maximum VIF = 3.655).
